# Evaluation of Left Ventricular Papillary Muscles Using Targeted Views by Echocardiography

**DOI:** 10.3390/jcm15093496

**Published:** 2026-05-02

**Authors:** Linyue Zhang, Yuji Xie, Xin Zhang, Yihan Chen, Yun Yang, He Li, Yuman Li, Mingxing Xie

**Affiliations:** 1 Department of Ultrasound Medicine, Union Hospital, Tongji Medical College, Huazhong University of Science and Technology, Wuhan 430022, China; d202582451@hust.edu.cn (L.Z.); xieyuji0323@hust.edu.cn (Y.X.); zxin1217@hust.edu.cn (X.Z.); yihan_chen@hust.edu.cn (Y.C.); yunyang97@hust.edu.cn (Y.Y.); lih0507@hust.edu.cn (H.L.); 2 Clinical Research Center for Medical Imaging in Hubei Province, Wuhan 430022, China; 3 Hubei Province Key Laboratory of Molecular Imaging, Wuhan 430022, China; 4 Key Laboratory of Biological Targeted Therapy (Huazhong University of Science and Technology), Ministry of Education, Wuhan 430022, China

**Keywords:** papillary muscles, echocardiography, targeted imaging, mitral valve, reference values, left ventricle, cardiac imaging

## Abstract

**Background**: Papillary muscles (PMs) are important for mitral valve competence and left ventricular mechanics, but accurate evaluation is often limited by poor visualization in conventional echocardiographic views. We developed papillary muscle–targeted (PM-targeted) echocardiographic views to improve PM visualization and aimed to validate this approach and establish normative reference values in healthy adults. **Methods**: In protocol 1, posteromedial papillary muscle (PPM) length and maximum diameter measured using PM-targeted and standard views were compared with anatomic measurements in ten ex vivo porcine hearts. In protocol 2, measurements of the anterolateral papillary muscle (APM) and PPM were compared between PM-targeted and standard views in 100 healthy adults. In protocol 3, PM structural, spatial, and functional parameters were measured using PM-targeted views in 245 healthy adults. In protocol 4, PM measurements obtained from 2D PM-targeted views were compared with 3D echocardiographic measurements in 50 patients with ventricular functional mitral regurgitation (VFMR); PM parameters in VFMR were also compared with those in healthy adults. **Results**: In protocol 1, PM-targeted views showed stronger correlation with anatomic measurements for PPM length than standard views (0.966 vs. 0.752, *p* = 0.049), while standard views underestimated PPM length. In protocol 2, PM-targeted views enabled complete visualization of APM and PPM and yielded longer PM lengths and smaller maximum diameters than standard views. In protocol 3, males had larger PM maximum diameters and longer tip-to-annulus distances than females (all *p* < 0.05). With aging, interpapillary distance reduction (ΔIPMD), IPMD fractional shortening (IPMD-FS), and APM length decreased, whereas end-systolic IPMD increased (all *p* < 0.05). PM parameters correlated positively with body surface area (all *p* < 0.05). In protocol 4, PM measurements obtained from 2D PM-targeted views showed no differences from 3D echocardiographic measurements and demonstrated good correlation and agreement across assessed PM parameters against 3D echocardiographic measurement as a standard reference. Compared with healthy adults, patients with VFMR showed altered PM geometry/remodeling patterns. **Conclusions**: PM-targeted echocardiographic views improve visualization and measurement of papillary muscles and provide normative reference values, facilitating more accurate evaluation of PM-related abnormalities in clinical practice.

## 1. Introduction

Accurate evaluation of the left ventricular papillary muscles (PMs)—including their size, spatial orientation, and structural integrity—is essential for understanding mechanisms of mitral valve dysfunction and guiding management in cardiomyopathies. Abnormal PM morphology contributes to leaflet tethering, systolic anterior motion, left ventricular outflow tract (LVOT) obstruction, and residual or recurrent mitral regurgitation (MR), all of which may influence surgical strategy and postoperative outcomes [1,2,3]. Consequently, reliable assessment of PM anatomy has direct clinical implications.

The anterolateral papillary muscle (APM) and posteromedial papillary muscle (PPM) are integral components of the mitral valve apparatus. Their displacement, hypertrophy, or anomalous insertion has been implicated in hypertrophic cardiomyopathy, dilated cardiomyopathy, ischemic heart disease, and functional MR [4,5,6,7]. Reflecting this relevance, current guidelines recognize that abnormalities of the mitral valve supporting apparatus may require adjunctive surgical procedures such as papillary muscle relocation or resection [8]. Accurate preoperative delineation of PM anatomy is therefore critical for optimal procedural planning [9,10,11,12].

Despite their clinical importance, PM abnormalities are frequently underrecognized preoperatively. Conventional echocardiography detects only a minority of PM anomalies, with many identified only during surgery. This limitation may contribute to incomplete repair and persistent postoperative MR or LVOT obstruction, highlighting the need for improved noninvasive imaging of PM anatomy.

Conventional two-dimensional echocardiography remains the most widely used modality for PM evaluation [13,14], but its fixed insonation planes are often misaligned with the obliquely oriented PMs, leading to foreshortening and inaccurate measurements. Although three-dimensional echocardiography [15,16,17] and cardiac magnetic resonance imaging [18,19,20] provide more comprehensive anatomical assessment, their routine clinical use may be limited by availability, image quality, cost, and patient-related factors [21].

To address these limitations, we developed a papillary muscle-targeted imaging approach. By systematically adjusting transducer position and insonation angle from standard transthoracic views, this method aims to achieve long-axis and orthogonal short-axis visualization of papillary muscles. In this study we sought to: (1) validate PM measurements obtained using PM-targeted views against anatomical reference standards in the ex vivo model; (2) compare PM measurements between PM-targeted and conventional echocardiographic views in healthy adults; (3) establish normative echocardiographic reference values for PM morphology, spatial relationships, and functional parameters across sex, age, and body size; (4) evaluate the agreement between PM measurements obtained from 2D PM-targeted views and 3D echocardiography in patients with ventricular functional mitral regurgitation (VFMR), and further compare PM parameters between patients with VFMR and healthy adults.

## 2. Methods

### 2.1. Subjects and Specimens

This study comprised four protocols conducted at Union Hospital, Tongji Medical College, Huazhong University of Science and Technology (Wuhan, China). Protocol 1 was conducted between September 2023 and October 2024 using ten fresh porcine hearts (weight range 200–300 g; mean 250 g) obtained from a local slaughterhouse to validate PM-targeted echocardiographic measurements against anatomic reference standards. The subsequent protocols involving human participants were initiated after clinical ethics approval was obtained on 26 September 2024; all participants provided written informed consent before enrollment. In protocol 2, 100 healthy adult volunteers (median age 37 [25, 50] years; 60 males) without cardiovascular disease were prospectively enrolled to compare papillary muscle measurements between PM-targeted and standard views; of 127 initially recruited participants, 27 were excluded due to suboptimal image quality. In protocol 3, 245 healthy adults stratified by age (18–40 years, n = 71; 41–64 years, n = 97; ≥65 years, n = 77) were analyzed to establish normative reference values. In protocol 4, 50 patients with moderate or greater ventricular functional mitral regurgitation were prospectively enrolled to evaluate the agreement between PM measurements obtained from two-dimensional PM-targeted views and three-dimensional echocardiography, and to compare PM parameters between patients with ventricular functional mitral regurgitation and healthy adults. 50 healthy volunteers were enrolled as a control group.

### 2.2. Clinical Data

Sex, age, height, weight, heart rate, and systolic/diastolic blood pressure were recorded. Body surface area (BSA) and body mass index (BMI) were calculated using standard formulas.

### 2.3. Echocardiographic Data

Standard transthoracic echocardiography was performed using an S5-1 transducer (1–5 MHz, EPIC 7, Philips Medical Systems, Andover, MA, USA). In protocol 1, ten fresh ex vivo porcine hearts were irrigated with saline, the ascending aorta clamped, and the left ventricle (LV) pressurized via the mitral valve to achieve physiological distension. The hearts were fixed in a water tank for imaging, and standard parasternal long-axis and papillary muscle-level short-axis views were obtained according to guidelines [22]. Because the APM was poorly visualized in parasternal views in ex vivo porcine hearts, measurement was limited to the PPM. Measurements included the papillary muscle length and mid-body maximum diameter. The parasternal long-axis PM-targeted view was optimized by transducer angulation to display the maximal PPM length, clearly delineating its base (LV wall origin) and apex (chordal insertion), enabling precise base-to-apex measurement. The short-axis PM-targeted view was adjusted to capture the minimal cross-sectional area at the PPM mid-body for maximum diameter assessment (Figure S1). After echocardiographic measurements were obtained, the porcine heart was opened along the midline of the anterior interventricular groove to expose the PPM for direct anatomical measurement (Figure S2).

**Figure 1:**
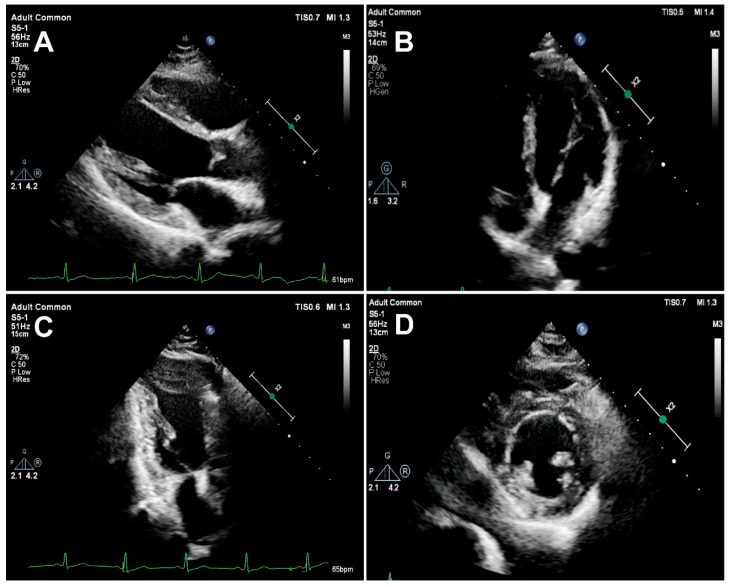
Schematic illustration of left ventricular papillary muscle targeted-view echocardiographic imaging. (**A**) Parasternal long-axis target view of the posteromedial papillary muscle (PPM); (**B**) apical four-chamber long-axis target view of the anterolateral papillary muscle (APM); (**C**) apical three-chamber long-axis target view of the PPM; (**D**) parasternal short-axis target view of the papillary muscles.

In protocol 2, transthoracic echocardiography was performed in healthy subjects according to American Society of Echocardiography guidelines [22], with images acquired over five consecutive cardiac cycles. Standard views included parasternal LV long- and short-axis views from the left sternal border and the apical four-, two-, and three-chamber views. In protocols 2 and 3, PM-targeted imaging was performed. For the APM: (1) apical four-chamber PM-targeted view: from the standard apical four-chamber, the probe was rotated ~20° counterclockwise to visualize the full APM from base (ventricular wall attachment) to apex (chordal insertion), ensuring complete long-axis imaging; (2) parasternal short-axis PM-targeted view: from the standard papillary muscle–level short-axis view, the probe was tilted to display the APM from base to apex, adjusting to minimize cross-sectional area and avoid oblique distortion. For the PPM: (1) parasternal long-axis PM-targeted view: from the standard LV long-axis, the probe was tilted toward the right ventricle (RV) inflow tract to display the entire PPM long axis from base to apex; (2) apical three-chamber PM-targeted view: from the standard apical three-chamber, the probe was slightly rotated clockwise to optimize long-axis visualization of PM; (3) parasternal short-axis PM-targeted view: from the papillary muscle–level short axis, the probe was tilted to capture the PPM from base to apex, with adjustments to obtain the smallest cross-section and avoid oblique cutting. Representative PM images are shown in Figure 1.

Conventional LV echocardiographic parameters were obtained according to the American Society of Echocardiography chamber quantification guidelines [23]. In protocol 2, LV PM length and maximum diameter were measured in both standard and PM-targeted views. End-diastolic lengths of the APM and PPM were obtained from standard parasternal LV long-axis and apical four-, two-, and three-chamber views, with the maximum value across each section recorded as the standard PM length. PM length was defined as the linear distance from the apical chordae insertion to the basal ventricular wall attachment. Mid-segment anteroposterior and left-right diameters at end diastole (outer edge to outer edge) were measured from the standard parasternal short-axis view at the PM level, and the larger of the two defined the PM maximum diameter [13,24]. In the PM-targeted views, APM length (base to apex) was measured in the apical four-chamber PM-targeted view. PPM length was measured in the parasternal and apical three-chamber PM-targeted views, with the larger value recorded. Mid-segment maximum diameters of both PMs were measured from the parasternal short-axis PM-targeted view, using the larger of the anteroposterior or lateral diameters. For PMs with multiple muscle columns, the two longest columns were averaged for length, and the maximum diameter of each column was used.

**Figure 2:**
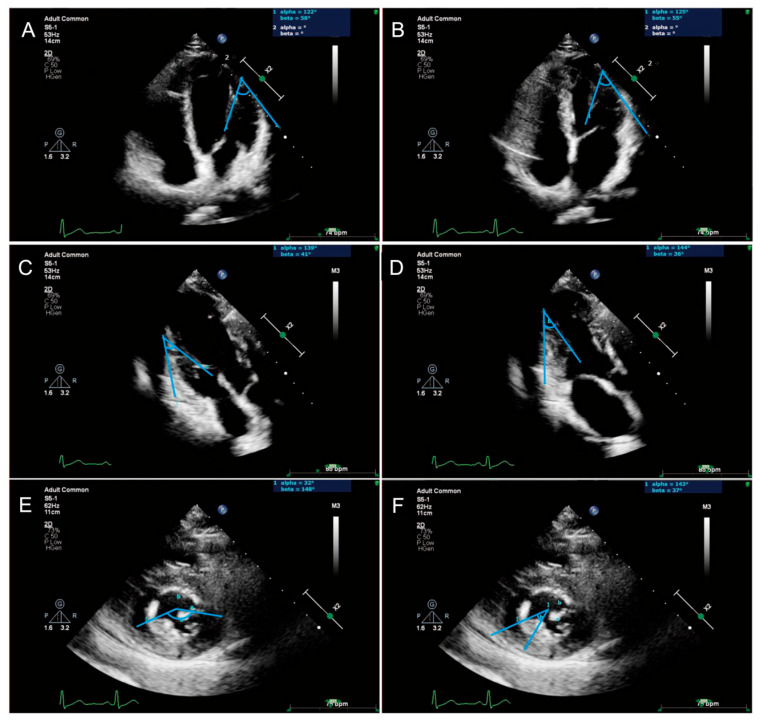
Representative echocardiographic images for left ventricular papillary muscle measurements. (**A**,**B**) Diastolic and systolic anterolateral papillary muscle (APM) with left ventricular wall angle; (**C**,**D**) diastolic and systolic posteromedial papillary muscle (PPM) with left ventricular wall angle; (**E**,**F**) systolic circumference angle of APM and PPM. APM, anterolateral papillary muscle; PPM, posteromedial papillary muscle.

**Figure 3:**
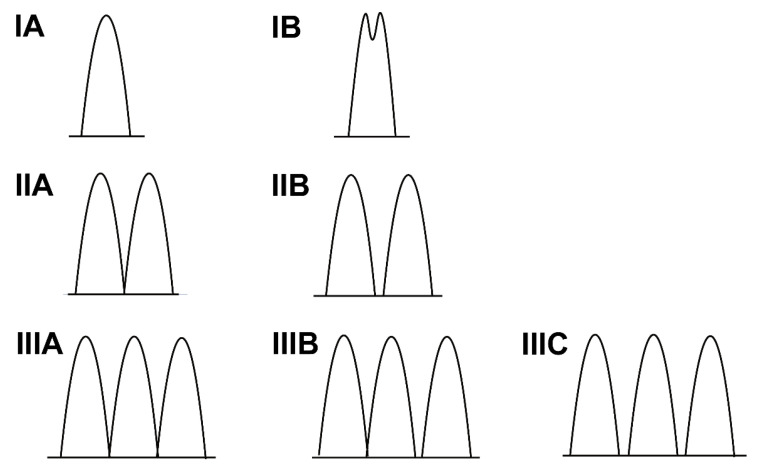
Schematic illustration of left ventricular papillary muscle morphological classification. IA, single muscle column without apical bifurcation; IB, single muscle column with apical bifurcation; IIA, two muscle columns with a common base; IIB, two muscle columns with separate bases; IIIA, three muscle columns with a common base; IIIB, three muscle columns, two of which share a common base; IIIC, three muscle columns with separate bases.

In protocol 3, detailed measurements of PM morphology were performed in the PM-targeted views. In a PM-targeted apical four-chamber view optimized for APM, end-diastolic (APM-Ld) and end-systolic lengths (APM-Ls) were measured. PPM lengths (PPM-Ld and PPM-Ls) were measured in the PM-targeted parasternal long-axis views. Fractional shortening (FS) was calculated for both muscles as FS = [(Ld − Ls)/Ld] × 100%. PM mobility was assessed as the angular difference between end-diastolic and end-systolic positions, and the vertical distance from the PM tip to the mitral annulus was measured during early and late systole. Basal-apical position was classified using the ratio of PM base-to-apex distance to mitral-annulus-to-apex distance: apical (<0.33), mid-ventricular (0.33–0.66), or basal (>0.66). In parasternal short-axis views, mid-body PM maximum diameter (APM-Md/PPM-Md) and circumferential angle relative to the LV cavity center were determined. Interpapillary distance dynamics were quantified as Interpapillary distance reduction (ΔIPMD) = Interpapillary muscle distance end-diastolic (IPMD_-ED_)—Interpapillary muscle distance end-systolic (IPMD_-ES_), and Interpapillary distance fractional shortening (IPMD-FS) = (ΔIPMD/IPMD_-ED_) × 100%. PM configurations were classified into three types: Type I, single column (IA: unbranched apex; IB: branched apex); Type II, bifid columns (IIA: shared base; IIB: separated bases); Type III, trifid columns (IIIA: common base; IIIB: two shared bases; IIIC: separated bases). An accessory papillary muscle was defined as a PM with an origin separate from the anterolateral and posteromedial papillary muscles [25], and the presence of accessory PMs was systematically documented. Comprehensive measurement protocols and classification criteria are illustrated in Figure 2 and Figure 3.

In protocol 4, transthoracic echocardiography was performed in patients with moderate or greater ventricular functional mitral regurgitation. Two-dimensional PM-targeted images and full-volume three-dimensional datasets were acquired at frame rates of 50–70 frames/s and 20–25 frames/s, respectively. Using multiplanar reconstruction, the three-dimensional datasets were sliced to define the long- and short-axis planes of each PM [26]. Measurements from the two-dimensional PM-targeted views and three-dimensional reconstruction were performed using the same definitions and measurement scheme as in protocol 3. In protocols 1–4, all measurements were averaged over three consecutive cardiac cycles to reduce the potential influence of beat-to-beat variability and heart rate fluctuation.

### 2.4. Reproducibility

Inter-observer and intra-observer reproducibility were evaluated across all protocols. Intra-observer variability was assessed by the same investigator who reanalyzed identical echocardiographic images after a 1-month interval. Inter-observer variability was assessed by a second investigator blinded to the initial measurements. This analysis was performed in 10 porcine hearts (Protocol 1), 15 randomly selected healthy adults (Protocol 2), and 30 randomly selected healthy adults (Protocol 3). All measurements were performed using an identical method to ensure methodological consistency.

### 2.5. Statistical Analysis

Continuous variables are presented as mean ± standard deviation (SD) for normally distributed data or as median (interquartile range) for non-normally distributed data, whereas categorical variables are expressed as frequencies and percentages.

In protocol 1, PM measurements among the standard views, PM-targeted views, and anatomical methods were compared using repeated-measures analysis of variance (ANOVA) followed by Bonferroni post hoc tests. In protocol 2, PM measurements obtained from the standard and PM-targeted views were compared using paired *t*-tests.

Reference intervals for echocardiographic papillary muscle parameters were established using a parametric approach. For variables with approximately normal distributions, the 95% reference intervals were calculated as mean ± 1.96 SD. Pearson or Spearman correlation coefficients were used, as appropriate, to assess correlations between PM measurements obtained from the PM-targeted and standard views (Protocols 1 and 2), correlations of PM measurements with age and body surface area (BSA) in healthy adults (Protocol 3), and correlations between PM measurements obtained from two-dimensional PM-targeted views and three-dimensional echocardiography (Protocol 4).

In protocol 4, paired *t*-tests were used to compare PM measurements obtained from two-dimensional PM-targeted views and three-dimensional echocardiography in patients with VFMR. Comparisons of PM parameters between patients with VFMR and healthy adults, as well as comparisons of clinical characteristics between included and excluded patients, were performed using the independent-samples *t*-test, Mann–Whitney U test, or chi-square test, as appropriate. Agreement was assessed using Bland–Altman analysis and intraclass correlation coefficients (ICCs).

All statistical analyses were performed using SPSS version 26.0 (IBM Corp., Armonk, NY, USA), GraphPad Prism version 9.5.1 (GraphPad Software, San Diego, CA, USA), and R version 4.1.1 (R Foundation for Statistical Computing, Vienna, Austria). A two-tailed *p* value < 0.05 was considered statistically significant.

## 3. Results

### 3.1. Protocol 1

Table S1 summarizes the PPM length and maximum diameter measured by the PM-targeted views, standard views, and anatomical gold-standard methods. There was no difference in PPM length or maximum diameter between PM-targeted views and anatomical measurements (*p* > 0.05), whereas standard views underestimated the PPM lengths compared with anatomical measurements (*p* < 0.05). For PPM maximum diameter, there was no difference between standard views and anatomical measurements (*p* > 0.05). The correlations of PM echocardiographic measurements with anatomical measurements are shown in Table S2. For PPM maximum diameter, measurements obtained from the PM-targeted views showed a correlation similar to that of measurements obtained from the standard views (0.927 vs. 0.926, *p* = 0.990). For PPM length, PM-targeted views measurements correlated more strongly than standard views measurements with anatomical measurements (0.966 vs. 0.752, *p* = 0.049).

For PPM length and maximum diameter, the bias and limits of agreement (LOA) between PM-targeted views and anatomical measurements were lower than the bias and LOA between standard views and anatomical measurements (Figure S3). Intra- and interobserver reproducibility was excellent for all PPM measurements, as evidenced by high ICCs, low bias and narrow LOA (Table S3).

### 3.2. Protocol 2

This study protocol included 100 healthy adults (60% male; median age 37.0 years) (Table S4). Visualization rates of left ventricular papillary muscles in standard echocardiographic views are shown in Table 1. The APM was not visualized in the parasternal long-axis or apical three-chamber views, seen in 42% of apical four-chamber and 25% of apical two-chamber views, and fully visualized in the parasternal short-axis views. PPM visualization rate ranged from 1% to 48% across standard long-axis and apical views, with 100% in the parasternal short-axis views. By contrast, PMs were clearly visualized in all PM-targeted echocardiographic views.

As illustrated in Figure S4, an echocardiographer who was already capable of independently performing adult transthoracic echocardiography, but had no prior experience with PM-targeted imaging, was able to acquire PM-targeted views in approximately 4 min per patient after completing around 33 consecutive cases.

The PM-targeted views yielded longer APM lengths in 57 subjects and longer PPM lengths in 91 subjects compared with standard views (Table 2). The PM-targeted views also yielded smaller APM and PPM maximum diameters across all 100 subjects compared with standard views (both *p* < 0.001). Correlation analysis showed strong associations for PM maximum diameters measured by the PM-targeted views and standard views (APM r = 0.935, *p* < 0.001; PPM r = 0.870, *p* < 0.001), with significantly stronger correlation for APM than PPM maximum diameter (0.935 vs. 0.870, *p* = 0.011). However, there was no correlation for APM length measurements between the PM-targeted views and standard views (r = −0.022, *p* = 0.872), and PPM length measured by the PM-targeted views had a weak correlation with that by the standard views (r = 0.293, *p* = 0.005) (Figure S5).

Bland–Altman analysis (Figure S6) indicated the bias and LOA for PM maximum diameters between the PM-targeted views and standard views were lower than that for PM lengths, indicating high agreement for PM maximum diameters but lower agreement for lengths.

Intra- and inter-observer reproducibility for PM measurements on the PM-targeted views and standard views are presented in Table S3. All measurements demonstrated good reproducibility and reliability, as evidenced by high ICCs, low bias, and narrow LOA.

### 3.3. Protocol 3

**Figure 4:**
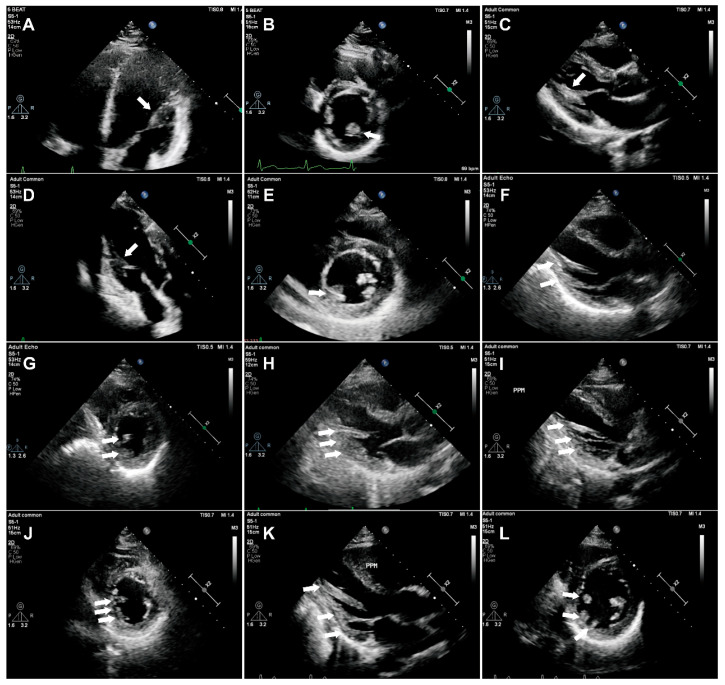
Echocardiographic images of left ventricular papillary muscle morphological classifications. (**A**,**B**) Long-axis and short-axis views of anterolateral papillary muscle (APM) IA type; (**C**) long-axis view of posteromedial papillary muscle (PPM) IB type; (**D**,**E**) long-axis and short-axis views of PPM IIA type; (**F**,**G**), long-axis and short-axis views of PPM IIB type; (**H**) long-axis view of PPM IIIA type; (**I**,**J**) long-axis and short-axis views of PPM IIIB type; (**K**,**L**) long-axis and short-axis views of PPM IIIC type.

This study protocol enrolled 245 healthy adults (130 males; 115 females) with a median age of 52 years (IQR 38–68) (Table S4). Of the 310 initially screened participants, 65 were excluded because of insufficient image quality for papillary muscle assessment, with suboptimal visualization of the papillary muscles mainly attributable to breast tissue interference (n = 33), excessive lung interference (n = 20), and narrow intercostal spaces (n = 12). As shown in Table S5, no significant differences in age, sex distribution, body mass index, body surface area, systolic blood pressure, diastolic blood pressure, or heart rate were observed between the included and excluded participants (all *p* > 0.05). Morphological analysis of PMs is summarized in Table 3 and Figure 4. APMs were predominantly Type IA (58.4%) and Type IIA (29.4%), while PPMs most frequently showed Type IIA (51.4%) and Type IIB (22.4%). Accessory PMs were present in 14 subjects (5.7%) (Figure S7). APMs mainly attached to the anterolateral and inferolateral LV walls; PPMs to the inferior wall. Both APMs and PPMs were primarily mid-ventricular (APM 97.1%; PPM 93.1%), with no basal attachments observed. Reference intervals for echocardiographic PM parameters are presented in Table 4.

Comparisons of PM parameters stratified by gender showed that males had larger APM and PPM maximum diameters and greater systolic tip-to-annulus distances than females (all *p* < 0.05), whereas the other PM parameters did not differ between males and females (Table 5). Comparisons of PM parameters stratified by age showed that subjects aged ≥ 41 years had lower interpapillary distance reduction (ΔIPMD), fractional shortening (IPMD-FS), and APM diastolic and systolic lengths than subjects aged 18–40 years (all *p* < 0.05). Subjects aged ≥ 65 years showed further reductions in ΔIPMD and IPMD-FS compared with those aged 41–64 years (*p* < 0.05), while other PM parameters were unaffected by age (Table 6).

Correlation analyses indicated that age inversely correlated with ΔIPMD (r = −0.444), IPMD-FS (r = −0.351), and APM lengths (r = −0.211 to −0.151), and positively correlated with IPMD_-ES_ (r = 0.231) (all *p* < 0.05). BSA positively correlated with APM/PPM maximum diameters (r = 0.266/0.170), APM lengths (r = 0.136 for APM-Ld, 0.155 for APM-Ls), and early and late systolic tip-to-annulus distances (APM r = 0.381, 0.397, respectively; PPM r = 0.469, 0.440, respectively) (all *p* < 0.05) (Table 7).

Intra- and interobserver reproducibility of PM parameters measured by the PM-targeted views was excellent (Table S3).

### 3.4. Protocol 4

The clinical and echocardiographic characteristics of the VFMR and control groups are presented in Table S6. A total of 50 patients with VFMR were included, with a median age of 59.5 years, of whom 24% were male. The control group consisted of 50 healthy volunteers, including 20 males and 30 females, with a median age of 57.0 years. No significant differences were observed in age, sex distribution, body mass index, body surface area or blood pressure between the two groups (all *p* > 0.05).

In patients with VFMR, 2D PM-targeted measurements were further compared with corresponding 3D echocardiographic measurements. No significant differences in PM diameters, PM lengths, PM–LV wall angles, or early systolic apex-to-mitral valve ring distances were found between the two methods (all *p* > 0.05; Table S7). Strong correlations for PM parameters were observed between 2D PM-targeted measurements and 3D measurements (r = 0.723–0.950, all *p* < 0.001) (Figure S8), and Bland–Altman analysis showed small biases with acceptable limits of agreement across all assessed PM parameters between the two methods (Figure S9).

IPMD-ED, IPMD-ES, and the APM diastolic end-to-left ventricular wall angle were significantly greater in the VFMR group than in the control group (all *p* < 0.05). In contrast, IPMD-FS, APM-FS, PPM-FS, the PPM diastolic end-to-left ventricular wall angle, and the PPM systolic end-to-left ventricular wall angle were significantly lower in the VFMR group than in the control group (all *p* < 0.05) (Table S6).

## 4. Discussion

To our knowledge, this study is among the first to establish and validate dedicated echocardiographic views specifically optimized for LV PM assessment. Using the PM-targeted views, we established normative echocardiographic reference values for PM morphology and motion in a healthy adult population and demonstrated the systematic influence of sex, age, and BSA on these parameters. The present findings are not intended to introduce a new set of routine echocardiographic measurements. Rather, they demonstrate that better alignment between imaging planes and papillary muscle anatomy can reduce geometric bias in conventional views and yield measurements that are more clinically interpretable when papillary muscle pathology is suspected.

### 4.1. PM-Targeted View Approach for Accurate Papillary Muscle Assessment

Conventional transthoracic echocardiographic views are optimized for assessment of global ventricular function and valvular morphology, rather than for elongated, variably oriented intracavitary structures such as PMs. As a result, PMs are frequently interrogated using oblique or foreshortened planes, leading to systematic underestimation of true PM length and overestimation of cross-sectional dimensions. The PM-targeted view approach was developed to address this limitation by dynamically adjusting probe position and insonation angle to achieve alignment with the individual long- and short-axis orientation of each PM.

Anatomical validation in ex vivo porcine hearts showed that PM-targeted views were more closely aligned with direct anatomic measurements of PPM length and maximum diameter than standard views, with lower measurement bias and narrower limits of agreement. This likely reflects improved alignment with the longitudinal axis of the PMs and more accurate orthogonal short-axis imaging, which reduce foreshortening and oblique sectioning. Although ex vivo models do not reproduce in vivo loading conditions, they provide a stable reference for evaluating the geometric accuracy of imaging planes, which was the primary objective of this validation.

### 4.2. PM Morphology and Reference Values in Healthy Adults

By applying the PM-targeted view method in a large cohort of healthy adults, the present study establishes comprehensive echocardiographic reference values for PM size, motion, and spatial relationships. The morphology and spatial arrangement of PMs are important determinants of left ventricular hemodynamics and mitral valve function, underscoring the clinical relevance of accurate anatomical assessment for surgical and interventional planning [14,27,28]. Consistent with prior gross anatomical and cardiac magnetic resonance imaging studies [2,18], we observed considerable interindividual variability in PM morphology, particularly with respect to the number of muscle heads and basal configuration. Accessory papillary muscles were identified in a small proportion of participants. This finding is clinically relevant, as prior studies have associated this anatomical variant with unexplained sudden cardiac arrest and procedural challenges during transcatheter edge-to-edge mitral valve repair, including limited device maneuverability and an increased risk of chordal entanglement [25,29]. These observations highlight the importance of systematic PM visualization for identifying both normal variants and potentially clinically relevant subvalvular anatomy.

PM hypertrophy is observed in conditions such as hypertension [1], hypertrophic cardiomyopathy [30], and Fabry disease [31], underscoring the clinical importance of accurate echocardiographic quantification for early disease screening. While prior studies have reported normal PM diameter using the standard short-axis view [13], measurements may be confounded by oblique sectioning in routine practice. In this study, PM diameter was measured using the targeted short-axis view, normal reference values were established, and significant associations with sex and body surface area were identified, providing a reliable anatomical reference for the assessment of PM hypertrophy in clinical and research settings.

PM insertion site also represents a key anatomical characteristic with clinical relevance. In this study, both APM and PPM were predominantly located at the mid-ventricular level in healthy adults, with no basal insertions observed. Because abnormal papillary muscle displacement contributes to left ventricular outflow tract obstruction and mitral regurgitation, particularly in hypertrophic cardiomyopathy [7,32], our findings indicate that mid-ventricular papillary muscle insertion represents the physiological pattern in healthy adults and may serve as a reference for identifying pathological subvalvular anatomy.

### 4.3. Papillary Muscle Dynamics and Geometric Relationships

Analysis of interpapillary muscle distance (IPMD) across the cardiac cycle revealed age-related differences in papillary muscle mechanics. Although absolute IPMD did not differ between sexes, increasing age was associated with reduced systolic shortening and a lower fractional change in IPMD, consistent with diminished centripetal papillary muscle motion. Previous studies have shown that impaired IPMD dynamics contribute to mitral leaflet tethering and ischemic mitral regurgitation independent of global ventricular size, highlighting the clinical relevance of these geometric measures even in the absence of overt ventricular dilation [33,34].

Papillary muscle orientation and displacement were additionally evaluated using circumferential and PM–LV wall angles. Kong [35] et al. used transthoracic echocardiography and a clock-face approach—defining the RV free wall–LV junction as zero—to measure angles to APM and PPM, which were significantly larger in patients with dilated cardiomyopathy than in controls. We considered that visualization of the right ventricular free wall–LV junction was often limited by acoustic windows, so circumferential angles were quantified relative to the LV cavity center. These angles effectively capture papillary muscle displacement, providing a sensitive metric for detecting geometric remodeling, particularly in dilated or hypertrophic cardiomyopathy.

Papillary muscle mobility was further assessed by measuring the angle between the PM and the LV wall. Patel et al. [20] quantitatively analyzed PM mobility in patients with hypertrophic cardiomyopathy using cardiac magnetic resonance (CMR) imaging and found that abnormal PM motion exceeding 10° was a reliable indicator of significant left ventricular outflow tract obstruction, highlighting the clinical significance of PM functional assessment.

Finally, the spatial relationship between the PM tip and the mitral annulus was evaluated. Komeda et al. [36] reported that the tip-to-annulus distance remains relatively stable throughout the cardiac cycle and under varying loading conditions, supporting its role as a reproducible surgical reference. This parameter is particularly relevant in mitral valve repair or replacement, where accurate estimation of chordal length and preservation of annulus–papillary muscle continuity are important to reduce the risk of postoperative left ventricular rupture [37]. In the present study, we provide normal reference values for this distance and observed that systolic tip-to-annulus length was greater in males and increased with body surface area, indicating that sex and body size may need to be considered during preoperative assessment and surgical planning. Overall, these findings provide normative reference ranges for papillary muscle morphology and geometry in healthy adults. Although some measurements varied significantly according to sex or body size, their clinical value in this setting lies primarily in establishing a structured anatomical reference against which disease-related abnormalities may be recognized and interpreted.

### 4.4. Clinical Significance of the PM-Targeted View Method

Accurate echocardiographic assessment of left ventricular papillary muscles (PMs) is increasingly relevant in mitral valve disease and other conditions in which PM geometry and motion affect intracardiac mechanics [1,38,39]. By improving alignment between the imaging plane and PM anatomy, the PM-targeted view may improve the consistency of PM measurements and provide a clearer anatomical basis for interpretation. Although three-dimensional echocardiography and cardiac magnetic resonance provide more comprehensive assessment of papillary muscle anatomy and the subvalvular apparatus, their routine use may be limited in daily practice. Our results demonstrate that the PM-targeted 2D approach is highly correlated with 3D measurements and exhibits good agreement. Therefore, the PM-targeted 2D approach is best understood as a focused adjunct to conventional transthoracic echocardiography that may improve papillary muscle visualization and allow more structured quantitative assessment when detailed PM evaluation is clinically relevant. Its main advantages are accessibility, ease of integration into routine echocardiographic workflow, and point-of-care applicability. PM-targeted 2D imaging may be considered a complementary technique with practical advantages in selected clinical settings, rather than a substitute for comprehensive advanced imaging.

In contrast to the healthy cohort, the clinical relevance of PM-related geometric measurements is more apparent in structural heart disease. In this small exploratory VFMR cohort, increased IPMD-_ED_ and IPMD-_ES_, together with reduced IPMD-FS, suggest greater papillary muscle separation and less systolic shortening. Lower APM-FS and PPM-FS further indicate impaired PM motion, whereas the altered PM–LV wall angles are consistent with remodeling of the subvalvular apparatus. In this setting, these differences are not only statistically significant, but also mechanistically meaningful, as papillary muscle displacement and altered tethering geometry are important features of functional mitral regurgitation [33,40].

Clinically, the PM-targeted view may add value by allowing more direct assessment of subvalvular remodeling in VFMR. This may be relevant not only for preprocedural evaluation and follow-up, but also for surgical planning when subvalvular distortion appears to contribute substantially to regurgitation. Particularly, subvalvular repair techniques such as papillary muscle approximation or relocation have been used to reduce leaflet tethering and restore mitral valve geometry in selected patients with functional mitral regurgitation [41,42,43]. In this setting, better delineation of PM position, motion, and its relationship to the LV wall may help clarify the mechanism of regurgitation and support a more individualized imaging assessment in routine practice.

### 4.5. Limitations and Future Directions

Several limitations should be acknowledged. First, this was a single-center study conducted in a single geographic region, which may limit the external generalizability of the proposed reference values. Second, a proportion of participants were excluded because of inadequate acoustic windows for papillary muscle assessment. Although the baseline clinical characteristics of included and excluded participants were comparable, potential selection bias related to image quality cannot be entirely excluded, and the feasibility of this approach may be reduced in subjects with suboptimal transthoracic windows. Third, anatomical validation was performed in ex vivo porcine hearts and therefore may not fully reflect in vivo human myocardial loading conditions. Finally, although normal reference values are appropriately derived from healthy individuals, the applicability of the PM-targeted view in structural heart disease requires further validation beyond the preliminary observations in VFMR.

Future studies should further assess the feasibility, reproducibility, and clinical utility of PM-targeted views in broader and disease-specific populations. With further refinement and multicenter validation, this targeted approach may serve as a useful adjunct for comprehensive subvalvular imaging in the selected clinical settings.

## 5. Conclusions

PM-targeted echocardiographic views may improve visualization and quantitative assessment of papillary muscles and provide normative reference values, thereby facilitating more accurate evaluation of PM-related abnormalities when detailed papillary muscle assessment is needed.

## Figures and Tables

**Table 1:** Visualization of PM in Standard Echocardiographic Views (n = 100).

Echocardiographic Standard View Type	APM Visualization Rate n (%)	PPM Visualization Rate n (%)
**Long-axis Views**		
Standard Parasternal Long-axis View of the Left Ventricle	0 (0.0)	34 (34.0)
Standard Apical Four-chamber View	42 (42.0)	1 (1.0)
Standard Apical Two-chamber View	25 (25.0)	48 (48.0)
Standard Apical Three-chamber View	0 (0.0)	30 (30.0)
**Short-axis Views**		
Standard Parasternal Papillary Muscle-level Left Ventricular Short-axis	100 (100.0)	100 (100.0)

The data are presented as frequency (percentage); PM, papillary muscle; APM, anterolateral papillary muscle; PPM, posteromedial papillary muscle.

**Table 2:** Comparisons of PM Measurements between Standard and PM-targeted Views.

	Variable	Standard View	PM-Targeted View	*p* Value
Length (mm)	APM (n = 57)	16.8 ± 2.9	28.9 ± 2.5	**<0.001**
	PPM (n = 91)	17.7 ± 2.8	27.3 ± 2.2	**<0.001**
Maximum diameter (mm)	APM (n = 100)	8.6 ± 0.9	8.5 ± 0.9	**<0.001**
	PPM (n = 100)	8.3 ± 1.0	8.1 ± 0.9	**<0.001**

The data are presented as mean ± standard deviation; PM, papillary muscle; APM, anterolateral papillary muscle; PPM, posteromedial papillary muscle.

**Table 3:** Morphological Characteristics of PM in Healthy Adults.

Parameters	APM (n = 245)	PPM (n = 245)
**Morphological Type, n (%)**		
IA	143 (58.4)	46 (18.8)
IB	7 (2.9)	11 (4.5)
IIA	72 (29.4)	126 (51.4)
IIB	20 (8.2)	55 (22.4)
IIIA	3 (1.2)	0 (0.0)
IIIB	0 (0.0)	4 (1.6)
IIIC	0 (0.0)	3 (1.2)
**Location Distribution, n (%)**		
Anterolateral	168 (68.6)	0 (0.0)
Inferolateral	77 (31.4)	0 (0.0)
Anterior	0 (0.0)	0 (0.0)
Inferior	0 (0.0)	245 (100.0)
Anteroseptal	0 (0.0)	0 (0.0)
Inferoseptal	0 (0.0)	0 (0.0)
**Longitudinal Position, n (%)**		
Basal Type	0 (0)	0 (0)
Midventricular Type	238 (97.1)	228 (93.1)
Apex Type	7 (2.9)	17 (6.9)
**Accessory Papillary Muscles, n (%)**	14 (5.7)	

The data are presented as frequency (percentage); PM, papillary muscle; APM, anterolateral papillary muscle; PPM, posteromedial papillary muscle.

**Table 4:** Reference Ranges for PM Measurements in Healthy Adults.

Parameters	Total (n = 245)	Male (n = 130)	Female (n = 115)
**Basic Dimensions**			
APM-Md (mm)	6.4–10.4	6.8–10.4	6.4–10.0
PPM-Md (mm)	6.3–9.9	6.6–9.8	6.1–9.7
APM-CA (°)	136.0–156.2	136.0–156.4	136.3–155.9
PPM-CA (°)	34.0–48.2	34.3–48.5	34.6–47.6
**Interpapillary Metrics**			
IPMD_-ED_ (mm)	18.5–25.1	18.5–25.1	18.9–25.3
IPMD_-ES_ (mm)	6.7–14.1	6.5–14.7	7.1–14.5
ΔIPMD (mm)	8.8–13.8	8.5–13.9	8.9–13.7
IPMD-FS (%)	39.1–65.3	37.3–65.9	39.2–63.8
**Length and Shortening**			
APM-Ld (mm)	21.8–34.8	21.7–34.7	21.7–34.3
APM-Ls (mm)	15.8–26.8	15.9–26.5	16.0–26.0
APM-FS (%)	14.8–38.4	14.1–38.7	15.8–37.8
PPM-Ld (mm)	24.9–30.9	23.8–32.0	24.1–31.5
PPM-Ls (mm)	18.5–23.9	17.8–24.4	17.9–24.5
PPM-FS (%)	18.3–29.7	21.0–27.6	15.7–31.7
**Angular Orientation** (°)			
APM Diastolic End with LV Wall	37.1–66.1	36.0–65.8	34.9–65.5
APM Systolic End with LV Wall	33.4–62.4	32.2–62.4	31.4–61.6
APM Mobility	2.4–5.2	2.0–5.2	2.1–5.3
PPM Diastolic End with LV Wall	31.6–53.2	31.1–53.9	33.5–51.1
PPM Systolic End with LV Wall	27.8–49.8	27.2–50.4	30.1–47.7
PPM Mobility	1.3–5.7	1.5–5.9	1.2–5.6
**Apex to Mitral Valve Ring Distance** (mm)			
*APM*			
Early Systolic	15.7–29.1	16.6–29.6	15.5–27.7
Late Systolic	14.7–29.5	15.4–30.2	15.5–27.3
*PPM*			
Early Systolic	15.8–30.8	17.5–30.9	13.8–31.0
Late Systolic	15.4–30.4	16.4–31.0	13.6–30.8

APM-Md, anterolateral papillary muscle maximum diameter; PPM-Md, posteromedial papillary muscle maximum diameter; APM-CA, anterolateral papillary muscle circumference angle; PPM-CA, posteromedial papillary muscle circumference angle; IPMD_-ED_, end-diastolic interpapillary muscle distance; IPMD_-ES_, end-systolic interpapillary muscle distance; ΔIPMD, interpapillary muscle distance reduction; IPMD-FS, interpapillary muscle distance fractional shortening; APM-Ld, anterolateral papillary muscle length at end-diastole; APM-Ls, anterolateral papillary muscle length at end-systole; APM-FS, anterolateral papillary muscle fractional shortening; PPM-Ld, posteromedial papillary muscle length at end-diastole; PPM-Ls, posteromedial papillary muscle length at end-systole; PPM-FS, posteromedial papillary muscle fractional shortening; PM, papillary muscle; APM, anterolateral papillary muscle; PPM, posteromedial papillary muscle.

**Table 5:** Comparisons of PM Measurements Stratified by Gender in Healthy Adults.

Parameters	Male (n = 130)	Female (n = 115)	*p* Value
**Basic Dimensions**			
APM-Md (mm)	8.6 ± 0.9	8.2 ± 0.9	**0.001**
PPM-Md (mm)	8.2 ± 0.8	7.9 ± 0.9	**0.003**
APM-CA (°)	146.2 ± 5.2	146.1 ± 4.9	0.878
PPM-CA (°)	41.4 ± 3.6	41.1 ± 3.3	0.577
**Interpapillary Metrics**			
IPMD_-ED_ (mm)	21.8 ± 1.7	22.1 ± 1.6	0.161
IPMD_-ES_ (mm)	10.6 ± 2.1	10.8 ± 1.9	0.562
ΔIPMD (mm)	11.2 ± 1.4	11.3 ± 1.2	0.354
IPMD-FS (%)	51.6 ± 7.3	51.5 ± 6.3	0.969
**Length and Shortening**			
APM-Ld (mm)	28.2 ± 3.3	28.0 ± 3.2	0.579
APM-Ls (mm)	21.2 ± 2.7	21.0 ± 2.5	0.421
APM-FS (%)	26.4 ± 6.3	26.8 ± 5.6	0.585
PPM-Ld (mm)	27.9 ± 2.1	27.8 ± 1.9	0.753
PPM-Ls (mm)	21.1 ± 1.7	21.2 ± 1.7	0.635
PPM-FS (%)	24.3 ± 1.7	23.7 ± 4.1	0.121
**Angular Orientation** (°)			
APM Diastolic End Angle	50.9 ± 7.6	50.2 ± 7.8	0.496
APM Systolic End Angle	47.3 ± 7.7	46.5 ± 7.7	0.448
APM Mobility	3.6 ± 0.8	3.7 ± 0.8	0.453
PPM Diastolic End Angle	42.5 ± 5.8	42.3 ± 4.5	0.749
PPM Systolic End Angle,	38.8 ± 5.9	38.9 ± 4.5	0.929
PPM Mobility	3.7 ± 1.1	3.4 ± 1.1	0.056
**Apex to MV Ring Distance** (mm)			
*APM*			
Early Systolic	23.1 ± 3.3	21.6 ± 3.1	**0.001**
Late Systolic	22.8 ± 3.8	21.4 ± 3.0	**0.003**
*PPM*			
Early Systolic	24.2 ± 3.4	22.4 ± 4.4	**0.006**
Late Systolic	23.7 ± 3.7	22.2 ± 4.4	**0.026**

Data are presented as mean ± standard deviation; APM-Md, anterolateral papillary muscle maximum diameter; PPM-Md, posteromedial papillary muscle maximum diameter; APM-CA, anterolateral papillary muscle circumference angle; PPM-CA, posteromedial papillary muscle circumference angle; IPMD_-ED_, end-diastolic interpapillary muscle distance; IPMD_-ES_, end-systolic interpapillary muscle distance; ΔIPMD, interpapillary muscle distance reduction; IPMD-FS, interpapillary muscle distance fractional shortening; APM-Ld, anterolateral papillary muscle length at end-diastole; APM-Ls, anterolateral papillary muscle length at end-systole; APM-FS, anterolateral papillary muscle fractional shortening; PPM-Ld, posteromedial papillary muscle length at end-diastole; PPM-Ls, posteromedial papillary muscle length at end-systole; PPM-FS, posteromedial papillary muscle fractional shortening; PM, papillary muscle; APM, anterolateral papillary muscle; PPM, posteromedial papillary muscle.

**Table 6:** Comparisons of PM Measurements Stratified by Age in Healthy Adults.

Parameters	18–40 Years (n = 71)	41–64 Years (n = 97)	≥65 Years (n = 77)	*p* Value
**Basic Dimensions**				
APM-Md (mm)	8.6 ± 1.1	8.3 ± 0.8	8.3 ± 0.9	0.059
PPM-Md (mm)	8.2 ± 1.0	8.1 ± 0.8	8.0 ± 0.8	0.159
APM-CA (°)	145.8 ± 5.2	146.9 ± 4.6	145.4 ± 5.4	0.121
PPM-CA (°)	41.4 ± 3.2	41.2 ± 3.4	41.2 ± 3.7	0.896
**Interpapillary Metrics**				
IPMD_-ED_ (mm)	21.9 ± 1.6	22.2 ± 1.7	21.6 ± 1.6	0.061
IPMD_-ES_ (mm)	9.9 ± 2.0	10.9 ± 1.9 ^a^	11.2 ± 1.9 ^a^	**<0.001**
ΔIPMD (mm)	12.0 ± 1.0	11.4 ± 1.3 ^a^	10.5 ± 1.0 ^ab^	**<0.001**
IPMD-FS (%)	55.1 ± 6.6	51.3 ± 6.5 ^a^	48.5 ± 6.0 ^ab^	**<0.001**
**Length and Shortening**				
APM-Ld (mm)	29.7 ± 3.3	27.4 ± 2.8 ^a^	27.6 ± 3.2 ^a^	**<0.001**
APM-Ls (mm)	22.2 ± 3.0	20.4 ± 2.1 ^a^	20.9 ± 2.4 ^a^	**<0.001**
APM-FS (%)	27.0 ± 6.3	27.1 ± 5.7	25.7 ± 6.1	0.279
PPM-Ld (mm)	28.0 ± 1.4	27.7 ± 2.1	27.8 ± 2.2	0.616
PPM-Ls (mm)	21.2 ± 1.4	21.1 ± 1.7	21.0 ± 1.9	0.829
PPM-FS (%)	24.1 ± 3.5	23.7 ± 3.1	24.4 ± 2.5	0.330
**Angular Orientation (°)**				
*APM*				
Diastolic End with LV Wall Angle	51.0 ± 9.1	50.7 ± 7.2	50.1 ± 6.8	0.759
Systolic End with LV Wall Angle	47.2 ± 9.1	46.9 ± 7.2	46.4 ± 6.8	0.725
Mobility	3.7 ± 0.7	3.8 ± 0.7	3.6 ± 0.8	0.425
*PPM*				
Diastolic End with LV Wall Angle	43.3 ± 5.8	43.3 ± 5.8	43.8 ± 6.5	0.794
Systolic End with LV Wall Angle	39.7 ± 6.0	39.7 ± 5.8	40.2 ± 6.5	0.822
Mobility	3.6 ± 1.1	3.6 ± 1.2	3.6 ± 1.0	0.836
**Apex to MV Ring Distance (mm)**				
*APM Early/Late Systolic*	21.8 ± 3.1/21.7 ± 3.1	22.4 ± 3.3/22.3 ± 3.3	22.9 ± 3.5/22.4 ± 4.1	0.154/0.466
*PPM Early/Late Systolic*	23.2 ± 4.7/22.9 ± 4.6	23.4 ± 3.2/23.0 ± 3.7	23.7 ± 4.1/23.3 ± 3.9	0.864/0.912

Data are presented as mean ± standard deviation; APM-Md, anterolateral papillary muscle maximum diameter; PPM-Md, posteromedial papillary muscle maximum diameter; APM-CA, anterolateral papillary muscle circumference angle; PPM-CA, posteromedial papillary muscle circumference angle; IPMD_-ED_, end-diastolic interpapillary muscle distance; IPMD_-ES_, end-systolic interpapillary muscle distance; ΔIPMD, interpapillary muscle distance reduction; IPMD-FS, interpapillary muscle distance fractional shortening; APM-Ld, anterolateral papillary muscle length at end-diastole; APM-Ls, anterolateral papillary muscle length at end-systole; APM-FS, anterolateral papillary muscle fractional shortening; PPM-Ld, posteromedial papillary muscle length at end-diastole; PPM-Ls, posteromedial papillary muscle length at end-systole; PPM-FS, posteromedial papillary muscle fractional shortening; PM, papillary muscle; APM, anterolateral papillary muscle; PPM, posteromedial papillary muscle; ^a^ *p* < 0.05 vs. 18–40 years; ^b^ *p* < 0.05 vs. 41–64 years.

**Table 7:** Correlations of PM Parameters with Age and BSA in Healthy Adults (n = 245).

Parameters		Age	BSA	
	*r*	*p* Value	*r*	*p* Value
**Basic Dimensions**				
APM-Md (mm)	−0.093	0.149	0.266	**<0.001**
PPM-Md (mm)	−0.065	0.312	0.170	**0.008**
APM-CA (°)	−0.01	0.875	0.059	0.361
PPM-CA (°)	−0.023	0.717	0.09	0.16
**Interpapillary Metrics**				
IPMD_-ED_ (mm)	−0.027	0.675	−0.098	0.128
IPMD_-ES_ (mm)	0.231	**<0.001**	−0.055	0.395
ΔIPMD (mm)	−0.444	**<0.001**	−0.066	0.303
IPMD-FS (%)	−0.351	**<0.001**	0.002	0.973
**Length and Shortening**				
APM-Ld (mm)	−0.211	**0.001**	0.136	**0.034**
APM-Ls (mm)	−0.151	**0.018**	0.155	**0.015**
APM-FS (%)	−0.001	0.993	−0.071	0.269
PPM-Ld (mm)	0.003	0.961	0.087	0.177
PPM-Ls (mm)	−0.014	0.823	0.011	0.87
PPM-FS (%)	0.039	0.540	0.085	0.186
**Angular Orientation** (°)				
APM Diastolic End with LV Wall Angle	−0.004	0.954	0.031	0.629
APM Systolic End with LV Wall Angle	0.006	0.925	0.033	0.611
APM Mobility	−0.03	0.642	−0.041	0.524
PPM Diastolic End with LV Wall Angle	0.103	0.109	−0.085	0.184
PPM Systolic End with LV Wall Angle	0.099	0.123	−0.093	0.147
PPM Mobility	0.017	0.793	0.029	0.649
**Apex to MV Ring Distance** (mm)				
APM Early Systolic	0.077	0.234	0.381	**<0.001**
APM Late Systolic	0.039	0.544	0.397	**<0.001**
PPM Early Systolic	0.039	0.633	0.469	**<0.001**
PPM Late Systolic	0.036	0.666	0.440	**<0.001**

APM-Md, anterolateral papillary muscle maximum diameter; PPM-Md, posteromedial papillary muscle maximum diameter; APM-CA, anterolateral papillary muscle circumference angle; PPM-CA, posteromedial papillary muscle circumference angle; IPMD_-ED_, end-diastolic interpapillary muscle distance; IPMD_-ES_, end-systolic interpapillary muscle distance; ΔIPMD, interpapillary muscle distance shortening value; IPMD-FS, papillary muscle distance fractional shortening; APM-Ld, anterolateral papillary muscle length at end-diastole; APM-Ls, anterolateral papillary muscle length at end-systole; APM-FS, anterolateral papillary muscle shortening fraction; PPM-Ld, posteromedial papillary muscle length at end-diastole; PPM-Ls, posteromedial papillary muscle length at end-systole; PPM-FS, posteromedial papillary muscle shortening fraction; PM, papillary muscle; APM, anterolateral papillary muscle; PPM, posteromedial papillary muscle; MV, mitral valve.

## Data Availability

The data supporting the findings of this study are available from the corresponding author upon reasonable request.

## Supplementary Materials

The following supporting information can be downloaded at: https://www.mdpi.com/article/10.3390/jcm15093496/s1, Table S1. Comparisons of PM Parameters among the PM-targeted Views, Standard Views, and Anatomical Measurements in Ex Vivo Porcine Hearts, Table S2. Correlations of PM Parameters on the PM-targeted and Standard Echocardiographic Views with Anatomical Measurements, Table S3. Intra-observer and inter-observer Reproducibility of PM parameters, Table S4. Clinical Characteristics and Left Ventricular Echocardiographic Parameters, Table S5. Comparison of clinical characteristics between included and excluded participants, Table S6. Clinical Characteristics and Papillary Muscle Parameters in the VFMR and Control Groups, Table S7. Comparison of Papillary Muscle Measurements Between the 2D PM-targeted View and 3D Echocardiography in Patients With VFMR, Figure S1. The echocardiographic images in an ex vivo porcine heart. Figure S2. Anatomical PPM measurements in an ex vivo porcine heart. Figure S3. Bland–Altman analysis of posteromedial papillary muscle measurements compared with anatomical measurements. Figure S4. Learning curve of obtaining transthoracic echocardiographic PM-targeted views. Figure S5. Correlations of papillary muscle measurements obtained between the target-view and standard-view methods. Figure S6. Bland–Altman analysis of papillary muscle measurements obtained with the target-view and standard-view methods. Figure S7. Echocardiographic images of the accessory papillary muscle. Figure S8. Correlations of papillary muscle measurements between 2D PM-targeted and 3D echocardiography views. Figure S9. Bland–Altman analysis of papillary muscle measurements between 2D PM-targeted and 3D echocardiography methods.
