# Supplementary material for: Evaluation of Left Ventricular Papillary Muscles Using Targeted Views by Echocardiography
Source: J Clin Med. 2026 May 2;15(9):3496. doi: 10.3390/jcm15093496 (PMC13163397; doi:10.3390/jcm15093496)
Supplement: Supplementary file 1 [file jcm-15-03496-s001.zip › jcm-4224731-supplementary.pdf]

Table S1. Comparisons of PM Parameters among the PM-targeted Views, Standard Views, and Anatomical Measurements in Ex Vivo Porcine Hearts (n = 10)

Parameters	Anatomical Measurements	PM-targeted Views	Standard Views	*P* value
PPM length (mm)	47 ± 4	47.3 ± 3.4	30.1 ± 5.4[ab]	< 0.001
PPM maximum diameter (mm)	25 ± 3	24.5 ± 4.0	26.0 ± 4.0[b]	0.006

Data are presented as mean ± standard deviation; PM, papillary muscle; PPM, posteromedial papillary muscle; [a] P < 0.05 compared with anatomical measured values; [b] P < 0.05 compared with targeted views.

Table S2. Correlations of PM Parameters on the PM-targeted and Standard Echocardiographic Views with Anatomical Measurements (n = 10)

	Parameters	*r* value	*P* value
PPM length (mm)	PM-targeted Views	0.966	< 0.001
	Standard Views	0.752	0.012
PPM maximum diameter (mm)	PM-targeted Views	0.927	< 0.001
	Standard Views	0.926	< 0.001

PM, papillary muscle; PPM, posteromedial papillary muscle.

Table S3. Intra-observer and inter-observer Reproducibility of PM parameters

Parameters	ICC (95%CI)	Bias	LOA
**Protocol 1 (n = 10)**			
Intra-observer			
Standard View PPM length (mm)	0.973 (0.897, 0.993)	-0.2	(-3.040 ~ 2.640)
Standard View PPM maximum diameter (mm)	0.989 (0.958, 0.997)	-0.17	(-1.332 ~ 0.992)
PM-targeted View PPM length (mm)	0.950 (0.812, 0.987)	-0.05	(-2.518 ~ 2.418)
PM-targeted View PPM maximum diameter (mm)	0.988 (0.955, 0.997)	0.21	(-1.007 ~ 1.427)
Inter-observer			
Standard View PPM length (mm)	0.971 (0.895, 0.993)	0.5	(-2.524 ~ 3.524)
Standard View PPM maximum diameter (mm)	0.985 (0.945, 0.996)	-0.28	(-1.668 ~ 1.108)
PM-targeted View PPM length (mm)	0.893 (0.624, 0.972)	0.02	(-2.938 ~ 2.978)
PM-targeted View PPM maximum diameter (mm)	0.987 (0.948, 0.997)	0.06	(-1.264 ~ 1.384)
**Protocol 2 (n = 15)**			
Intra-observer			
Standard View APM Length (mm)	0.902 (0.741, 0.966)	-0.33	(-2.462 ~ 1.808)
Standard View PPM Length (mm)	0.899 (0.706, 0.966)	-0.61	(-2.987 ~ 1.774)
PM-targeted View APM Length (mm)	0.952 (0.661, 0.987)	0.627	(-0.662 ~ 1.915)
PM-targeted View PPM Length (mm)	0.821 (0.530, 0.937)	0.473	(-1.365 ~ 2.311)
Inter-observer			
Standard View APM Length (mm)	0.948 (0.447, 0.988)	-0.67	(-1.773 ~ 0.427)
Standard View PPM Length (mm)	0.858 (0.633, 0.950)	-0.29	(-3.504 ~ 2.930)
PM-targeted View APM Length (mm)	0.915 (0.759, 0.971)	0.52	(-1.747 ~ 2.787)
PM-targeted View PPM Length (mm)	0.816 (0.539, 0.934)	-0.17	(-2.200 ~ 1.853)
**Protocol 3 (n = 30)**			
Intra-observer			
APM-CA (°)	0.965 (0.925, 0.983)	-0.89	(-5.968 ~ 4.181)
PPM-CA (°)	0.961 (0.921, 0.981)	-0.28	(-3.443 ~ 2.890)
APM-Md (mm)	0.955 (0.867, 0.981)	0.19	(-0.426 ~ 0.806)
PPM-Md (mm)	0.938 (0.868, 0.971)	0.14	(-0.589 ~ 0.869)
APM-Ld (mm)	0.990 (0.979, 0.995)	-0.003	(-0.766 ~ 0.759)
PPM-Ld (mm)	0.943 (0.886, 0.973)	0.11	(-1.448 ~ 1.668)
Inter-observer			
APM-CA (°)	0.968 (0.928, 0.985)	0.977	(-3.813 ~ 5.767)
PPM-CA (°)	0.992 (0.983, 0.996)	0.123	(-1.279 ~ 1.526)
APM-Md (mm)	0.939 (0.877, 0.971)	0.043	(-0.749 ~ 0.835)
PPM-Md (mm)	0.874 (0.752, 0.938)	-0.03	(-1.104 ~ 1.037)
APM-Ld (mm)	0.937 (0.873, 0.969)	0.227	(-1.863 ~ 2.316)
PPM-Ld (mm)	0.880 (0.754, 0.942)	-0.45	(-2.845 ~ 1.939)

PM, papillary muscle; ICC, intraclass correlation coefficient; CI, confidence interval; Bias, bias value; LOA, limits

of agreement; PPM, posteromedial papillary muscle; APM, anterolateral papillary muscle; APM-CA, anterolateral papillary muscle circumference angle; PPM-CA, posteromedial papillary muscle circumference angle; APM-Md, anterolateral papillary muscle maximum diameter; PPM-Md, posteromedial papillary muscle maximum diameter; APM-Ld, anterolateral papillary muscle length at end-diastole; PPM-Ld, posteromedial papillary muscle length at end-diastole

Table S4. Clinical Characteristics and Left Ventricular Echocardiographic Parameters

Variables	Protocol 2 (n=100)	Protocol 3 (n=245)	
**Clinical characteristics**			
Age (years)	37.0(25.3, 49.6)		51.8(37.8, 68.4)
		18 ~ 40 years, n (%)	71(29.0)
		41 ~ 64 years, n (%)	97(39.6)
		≥65 years, n (%)	77(31.4)
Male, n (%)	60(60.0)		130(53.1)
BMI (kg/m^2)	22.2 ± 2.6		22.3 ± 2.6
BSA (m^2)	1.8 ± 0.2		1.8(1.6, 1.9)
SBP (mmHg)	122.0(112.3, 130.0)		123.0(117.0, 130.0)
DBP (mmHg)	80.1 ± 9.9		79.0(73.0, 85.0)
HR (bpm)	75.0(67.3, 80.0)		73(65, 80)
**Left Ventricular Echocardiographic Parameters**			
IVST (mm)	9.0(8.7, 9.6)		9.1 ± 0.8
LVPWT (mm)	9.0(8.5, 9.5)		9.1(8.6, 9.6)
LVEDD (mm)	44.7(42.9, 47.2)		45.2 ± 3.4
LVESD (mm)	25.3(23.1, 27.0)		27.3(24.7, 30.2)
LVEF (%)	63.0(61.0, 67.0)		61.0(60.0, 63.0)
LVM (g)	132.9(117.1, 147.7)		134.7(116.9, 149.6)
LVMI (g/m^2)	72.9(66.6, 83.1)		75.5(67.3, 83.8)

The data are presented as mean ± standard deviation, median (interquartile range), or frequency (percentage); BMI, body mass index; BSA, body surface area; SBP, systolic blood pressure; DBP, diastolic blood pressure; HR, heart rate; IVST, interventricular septal thickness; LVPWT, left ventricular posterior wall thickness; LVEDD, left ventricular end-diastolic dimension; LVESD, left ventricular end-systolic dimension; LVEF, left ventricular ejection fraction; LVM, left ventricular mass; LVMI, left ventricular mass index.

Table S5. Comparison of clinical characteristics between included and excluded participants

Variable	Included participants (n = 245)	Excluded participants (n = 65)	*P* value
Age(years)	51.8(37.8, 68.4)	53.9(41.8, 66.4)	0.691
Male, n(%)	130(53.1)	32(52.5)	0.933
BMI (kg/m²)	22.1(20.5, 23.9)	21.9(20.5, 24.4)	0.832
BSA (m²)	1.8(1.6, 1.9)	1.8(1.6, 1.9)	0.711
SBP (mmHg)	123.0(117.0, 130.0)	120(112.5, 130.0)	0.165
DBP (mmHg)	79.0(73.0, 85.0)	79.0(73.0, 85.0)	0.304
HR (bpm)	73(65, 80)	75(66, 80)	0.497

The data are presented as mean ± standard deviation, median (interquartile range), or frequency (percentage); BMI, body mass index; BSA, body surface area; SBP, systolic blood pressure; DBP, diastolic blood pressure; HR, heart rate

Table S6. Clinical Characteristics and Papillary Muscle Parameters in the VFMR and Control Groups

Variable	VFMR Group (n = 50)	Control Group (n = 50)	P value
Age (years)	59.5(53.0, 65.0)	57.0(48.0, 62.3)	0.147
Male, n(%)	12(24)	20(40)	0.086
BMI (kg/m²)	22.9(21.3, 25.3)	22.3(20.6, 23.8)	0.114
BSA (m²)	1.8 ± 0.1	1.8±0.2	0.401
SBP (mmHg)	119.0(111.8, 127.0)	118.0(115.0, 121.0)	0.351
DBP (mmHg)	72.5(64.0, 79.3)	74.0(71.0, 77.3)	0.185
IPMD-ED (mm)	25.1(21.0, 28.7)	21.9(20.4, 23.4)	< 0.001
IPMD-ES (mm)	19.7(15.5, 22.6)	10.6(8.6, 12.6)	< 0.001
IPMD-FS (%)	23.5±6.7	55.4±7.3	< 0.001
APM-FS (%)	25.0±5.8	28.5±6.2	0.004
PPM-FS (%)	15.9±3.9	24.0±3.2	< 0.001
APM Diastolic End with LV Wall Angle (°)	56.0(51.0, 60.3)	51.2(44.8, 59.7)	0.015
APM Systolic End with LV Wall Angle (°)	50.0(45.8, 56.0)	47.8(41.1, 56.3)	0.090
PPM Diastolic End with LV Wall Angle (°)	38.0(33.0, 43.3)	42.0(40.0, 47.0)	0.004
PPM Systolic End with LV Wall Angle (°)	34.0(29.0, 39.3)	38.9(35.0, 43.1)	0.002

Data are expressed as the mean ± standard deviation, number (%), or as the median (interquartile range).VFMR, ventricular functional mitral regurgitation; BMI, body mass index; BSA, body surface area; SBP, systolic blood pressure; DBP, diastolic blood pressure; IPMD-ED, end-diastolic interpapillary muscle distance; IPMD-ES, end-systolic interpapillary muscle distance; IPMD-FS, interpapillary muscle distance fractional shortening; APM-FS, anterolateral papillary muscle fractional shortening; PPM-FS, posteromedial papillary muscle fractional shortening; PM, papillary muscle; APM, anterolateral papillary muscle; PPM, posteromedial papillary muscle.

Table S7. Comparison of Papillary Muscle Measurements Between the 2D PM-targeted View and 3D Echocardiography in Patients with VFMR (n = 50)

Variable	2D PM-targeted View	3D echocardiography	P value
APM-Md (mm)	8.1 ± 0.7	8.1 ± 0.6	0.861
PPM-Md (mm)	7.8 ± 0.9	7.9 ± 0.7	0.194
APM-Ld (mm)	28.1 ± 3.1	28.3 ± 2.8	0.388
PPM-Ld (mm)	27.7 ± 4.1	28.1 ± 3.2	0.132
APM Diastolic End with LV Wall Angle (°)	56.7 ± 7.7	56.8 ± 7.1	0.598
PPM Diastolic End with LV Wall Angle (°)	39.7 ± 8.1	39.6 ± 8.5	0.777
Early Systolic APM Apex to Mitral Valve Ring Distance (mm)	23.8 ± 2.3	24.0 ± 2.3	0.281
Early Systolic PPM Apex to Mitral Valve Ring Distance (mm)	24.9 ± 2.3	25.1 ± 2.2	0.191

Data are presented as mean ± standard deviation; 2D, two-dimensional; 3D, three-dimensional; VFMR, ventricular functional mitral regurgitation; APM-Md, anterolateral papillary muscle maximum diameter; PPM-Md, posteromedial papillary muscle maximum diameter; APM-Ld, anterolateral papillary muscle length at end-diastole; PPM-Ld, posteromedial papillary muscle length at end-diastole; PM, papillary muscle; APM, anterolateral papillary muscle; PPM, posteromedial papillary muscle

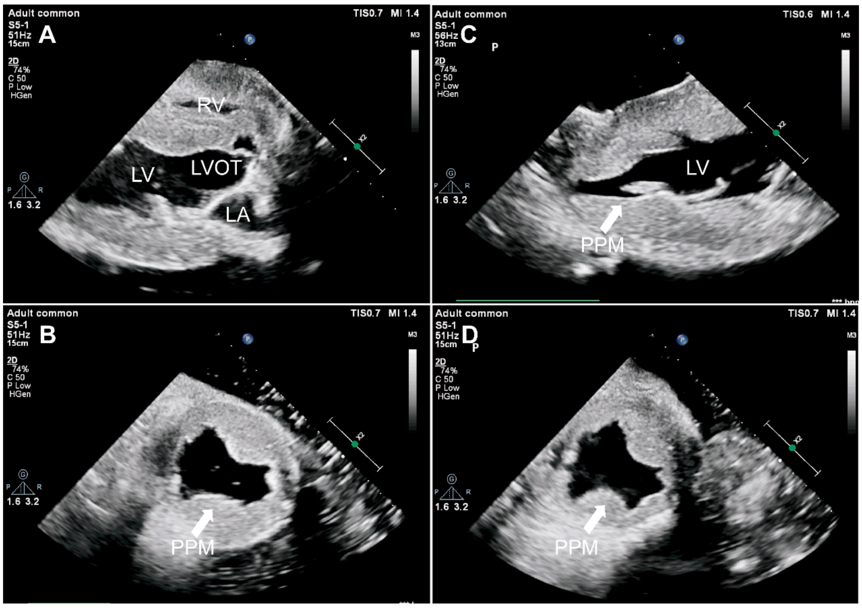

Figure S1. The echocardiographic images in an ex vivo porcine heart. (A) Standard parasternal long-axis view of the left ventricle; (B) Standard parasternal short-axis view of the LV at the level of the papillary muscles; (C) Parasternal long-axis targeted view of the posterior medial papillary muscle (PPM); (D) Parasternal short-axis targeted view of the PPM. RV, right ventricle; LV, left ventricle; LVOT, left ventricular outflow tract; LA, left atrium; PPM, posterior medial papillary muscle.

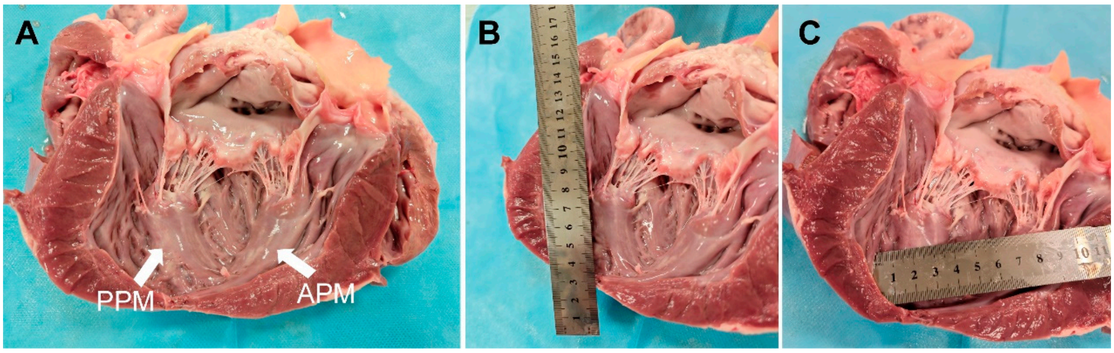

Figure S2. Anatomical PPM measurements in an ex vivo porcine heart. (A) Anatomical diagram of the left ventricular papillary muscles in the porcine heart; (B) Measurement of posterior medial papillary muscle (PPM) length; (C) Measurement of PPM maximum diameter. APM, anterolateral papillary muscle.

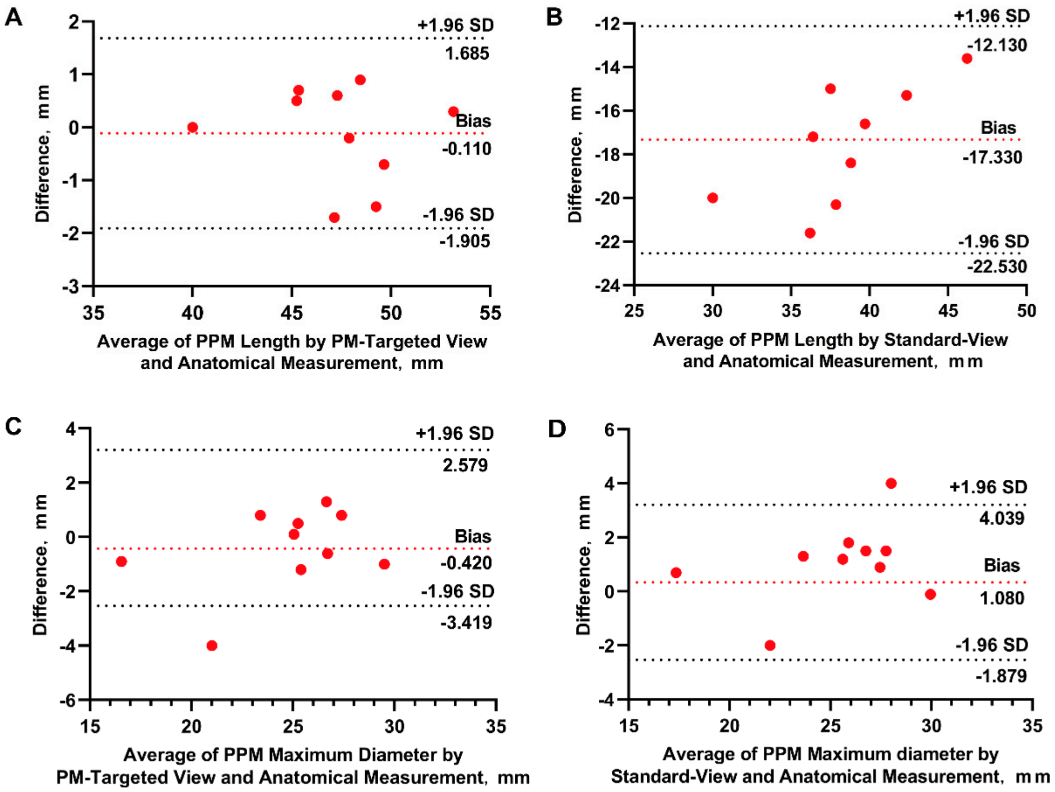

Figure S3. Bland–Altman analysis of posteromedial papillary muscle measurements compared with anatomical measurements. A, PPM length (A) and maximum diameter (C) between the PM-targeted view and anatomical measurements; B, PPM length (B) and maximum diameter (D) between the standard view and anatomical measurements. PPM, posteromedial papillary muscle.

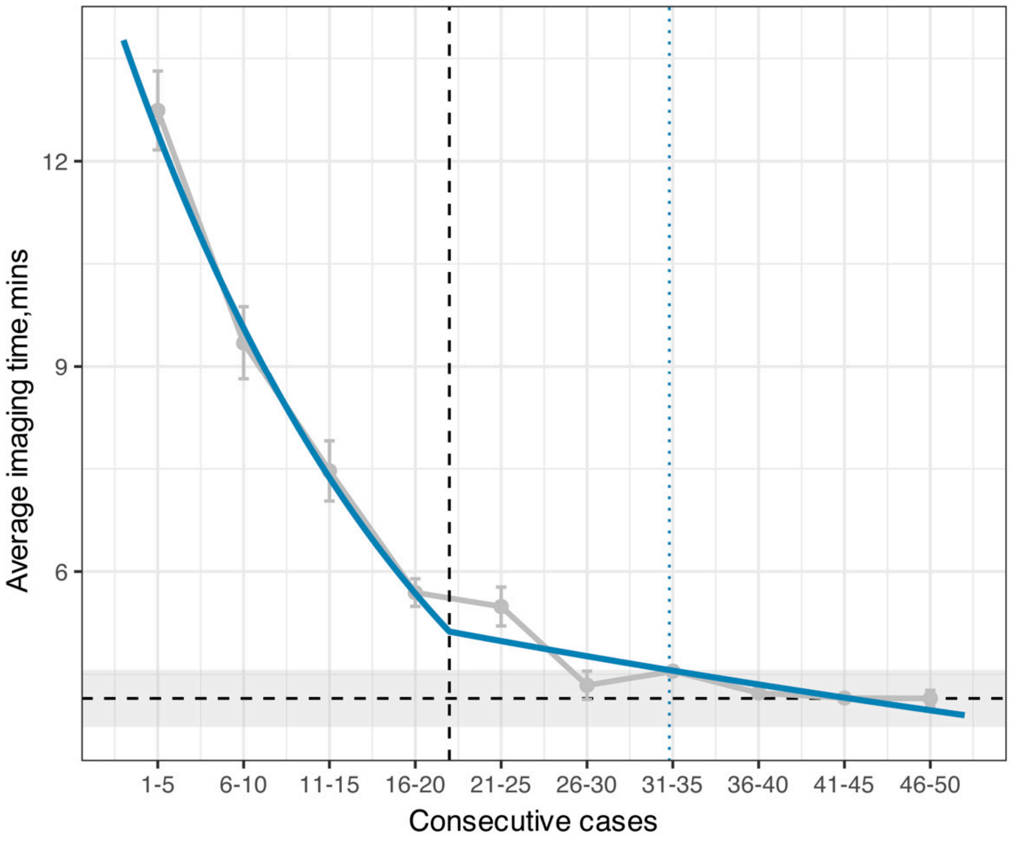

Figure S4. Learning curve of obtaining transthoracic echocardiographic PM-targeted views. The X axis refers to the first 50 consecutive study patients imaged by a single echocardiographer. The Y axis presents the average time spent acquiring the PM-targeted views per five consecutive cases.

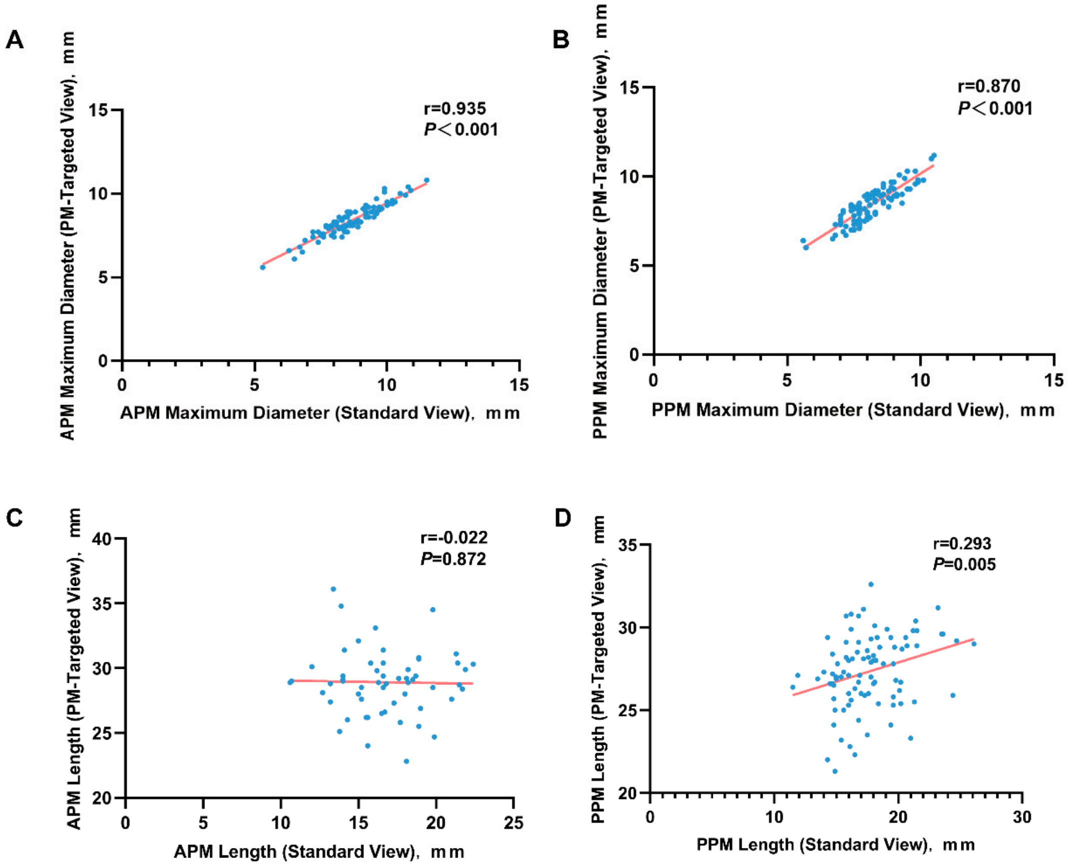

Figure S5. Correlations of papillary muscle measurements obtained between the PM-targeted view and standard-view methods. Strong correlation for anterolateral papillary muscle (APM) maximum diameter (A), strong correlation for posteromedial papillary muscle (PPM) maximum diameter (B), no significant correlation for APM length (C), and weak correlation for PPM length (D) between the target-view and standard-view methods. APM, anterolateral papillary muscle; PPM, posteromedial papillary muscle.

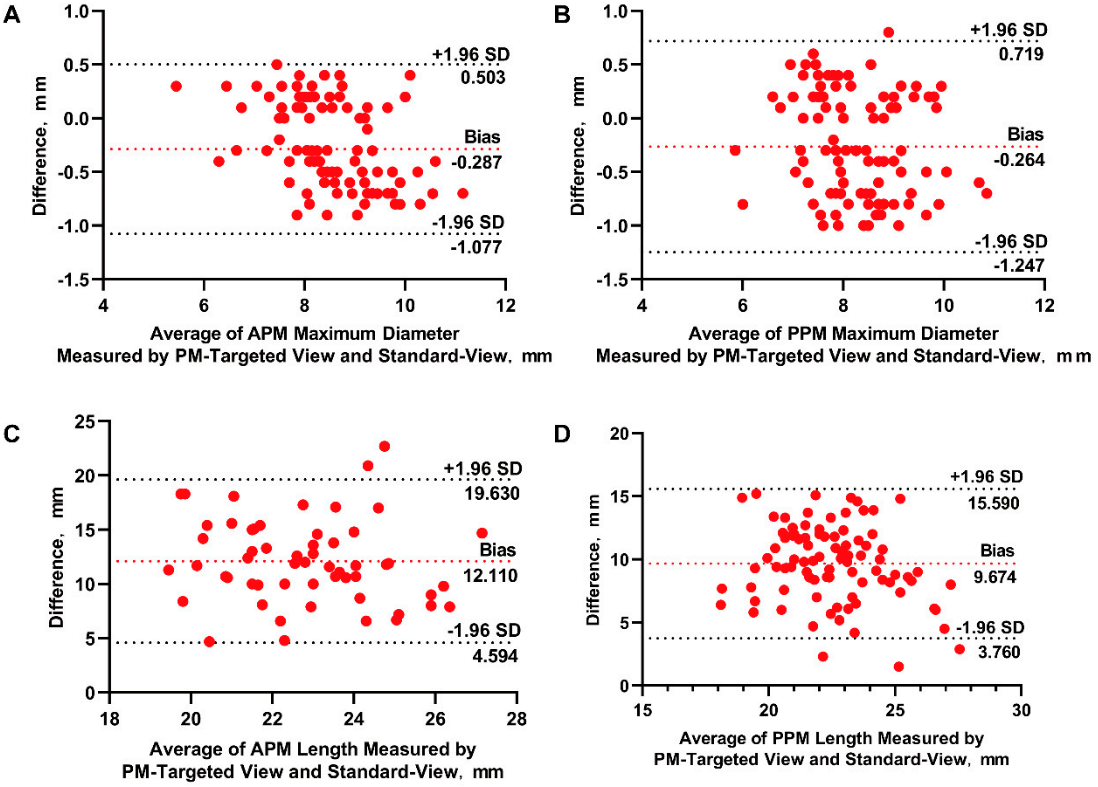

Figure S6. Bland–Altman analysis of papillary muscle measurements obtained with the PM-targeted view and standard-view methods. Bland–Altman analysis of anterolateral papillary muscle (APM) maximum diameter (A), posteromedial papillary muscle (PPM) maximum diameter(B), APM length (C), PPM length (D) between the target-view and standard-view methods. APM, anterolateral papillary muscle; PPM, posteromedial papillary muscle.

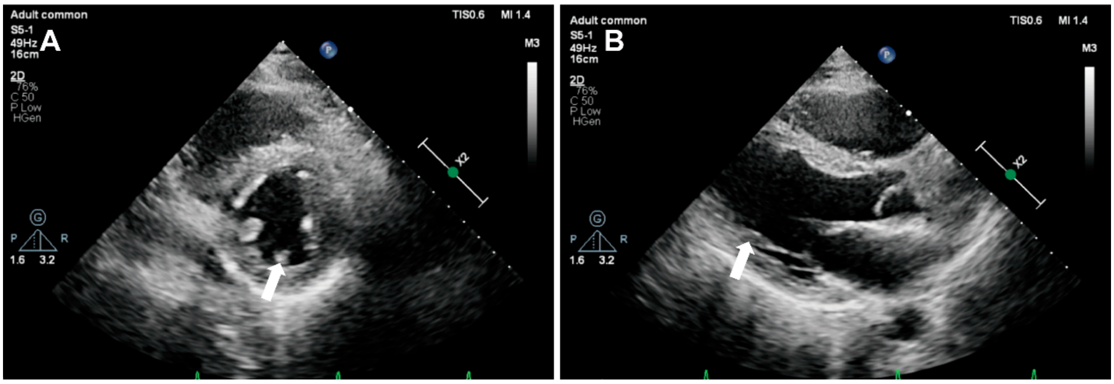

Figure S7. Echocardiographic images of the accessory papillary muscle. A, short-axis view; B, long-axis view. White arrows indicate the accessory papillary muscle.

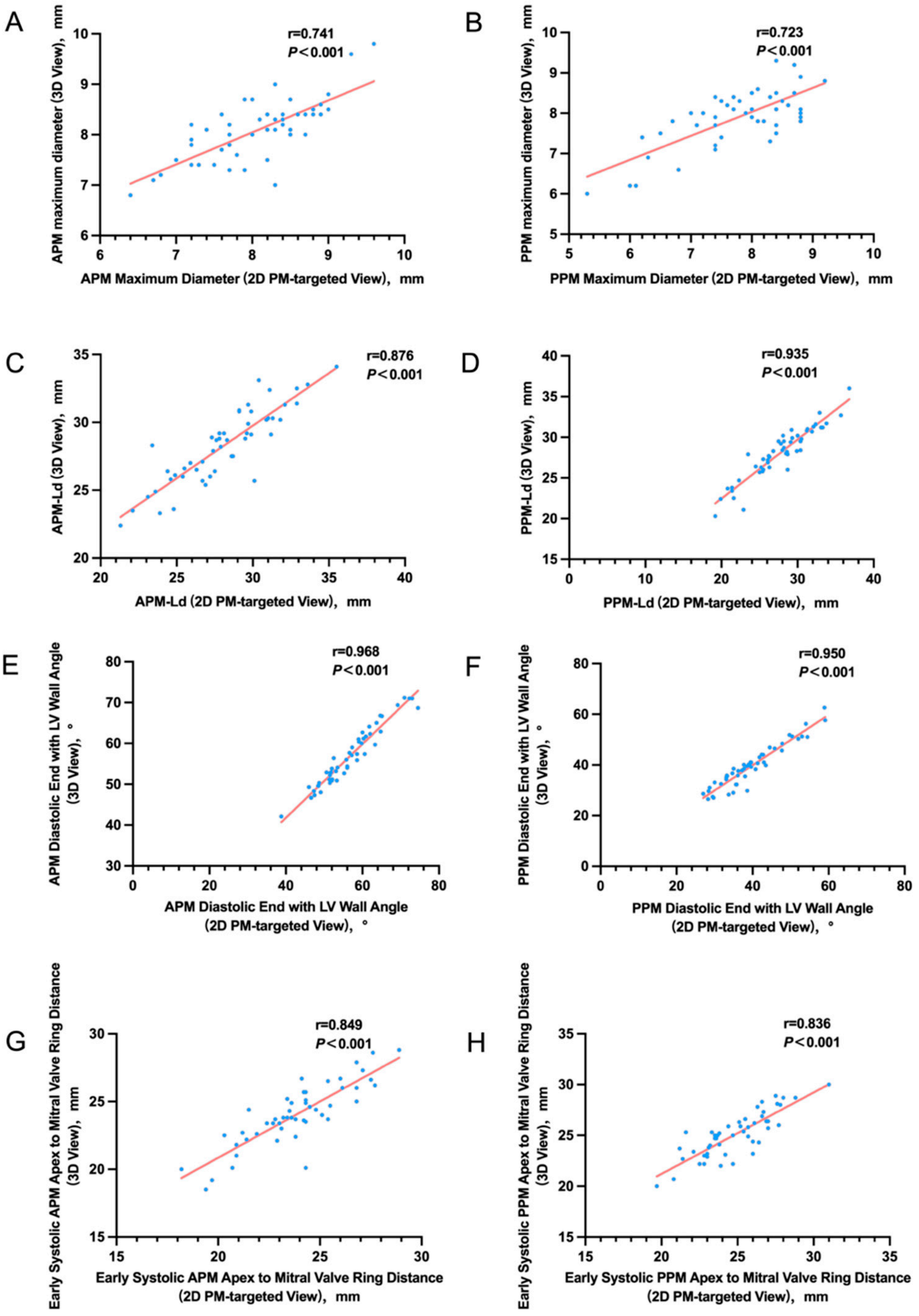

Figure S8. Correlations of papillary muscle measurements between 2D PM-targeted views and 3D echocardiography. Subplots A–H show correlations for APM maximum diameter (A), PPM maximum diameter (B), APM length (C), PPM length (D), APM diastolic end-to-LV wall angle (E), PPM diastolic end-to-LV wall angle (F), early systolic APM apex-to-mitral valve ring distance (G), and early systolic PPM apex-to-mitral valve ring distance (H) between the both methods. APM, anterolateral papillary muscle; PPM, posteromedial papillary muscle; Ld, length at end-diastole; LV, left ventricle.

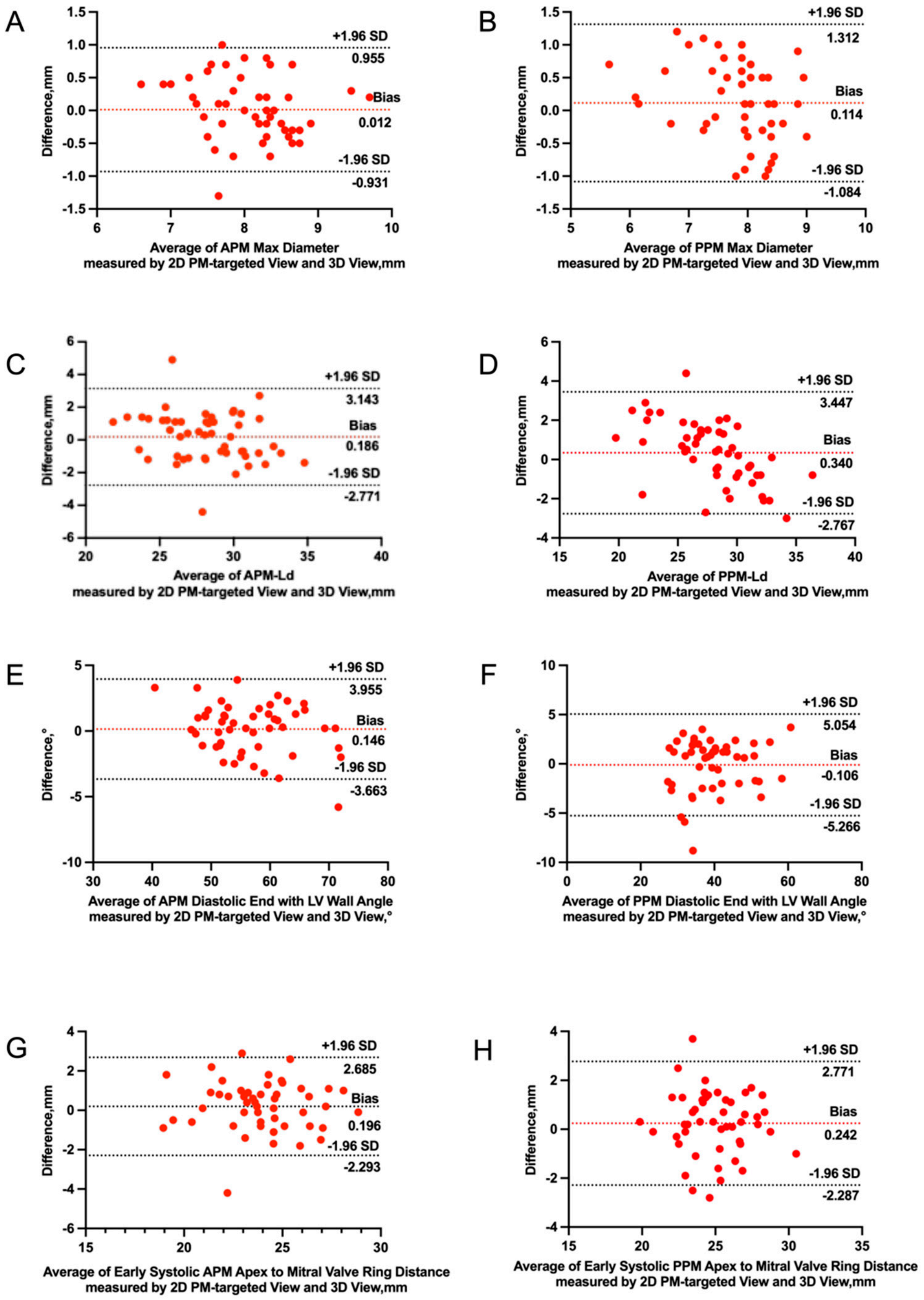

Figure S9. Bland–Altman analysis of papillary muscle measurements between 2D PM-targeted and 3D echocardiography methods. A–H show Bland–Altman analyses for APM maximum diameter (A), PPM maximum diameter (B), APM length (C), PPM length (D), APM diastolic end-to-LV wall angle (E), PPM diastolic end-to-LV wall angle (F), early systolic APM apex-to-mitral valve ring distance (G), and early systolic PPM apex-to-mitral valve ring distance (H) between the both methods. APM, anterolateral papillary muscle; PPM, posteromedial papillary muscle; Ld, length at end-diastole; LV, left ventricle.
